# Liquid phytase as a complete substitute for supplemental inorganic phosphorus in plant-based diets for commercially farmed Nile tilapia (*Oreochromis niloticus*)

**DOI:** 10.1186/s12917-026-05625-2

**Published:** 2026-07-23

**Authors:** Abdel-Fattah M. El-Sayed, Salma M.S. Zeid, Emad Afifi, Khaled Mahmoud, Nermin E. Khafaji, Sarah O. Makled

**Affiliations:** 1 Oceanography Department, Faculty of Science, Alexandria University, Alexandria, Egypt; 2 Aquaculture Technical Expert, Tanta, Egypt

**Keywords:** Nile tilapia, Phytase, Performance, Profitability, Phosphorus retention, Immunity

## Abstract

This research project addressed the effect of commercial, liquid phytase (Ronozyme^®^HiPhos- 20,000 FYT g^− 1^) (dsm-firmenich, Switzerland), produced by *Aspergillus niger*, on growth performance, economic return, enzymatic activities, and immune defense in Nile tilapia (*Oreochromis niloticus*) raised in a commercial tilapia farm. Adult, all-male fish, weighing 75.28 ± 0.66 g on average, were housed in triplicate in 2-m^3^ hapas, placed in an earthen fishpond, at a density of 40 fish hapa^− 1^. The fish were offered a basal commercial, extruded (floating) tilapia diet (3 mm diameter, 30% CP and 18.6 MJ kg^− 1^), supplemented with 10 g monocalcium phosphate (MCP) kg^− 1^ (control), 2000 FYT phytase kg^− 1^ or 50% of both MCP and phytase (5 g MCP + 1000 FYT kg^− 1^) for 80 days. performance, muscle composition, and the activities of digestive and liver enzymes were not affected (*P* > 0.05) in all treatments. Phosphorus (P) content in fish flesh and bones significantly increased in individuals fed phytase-based diets compared to the MCP-based feed. Phytase-based diets at 2000 FYT kg^− 1^ (with no MCP supplementation) and 1000 FYT kg^− 1^ plus 5 g MCP kg^− 1^ were more profitable than the reference diet. Exogenous phytase significantly increased (*p* < 0.05) blood erythrocytes (RBCs), hemoglobin (Hb), and leukocytes (WBCs), while mean corpuscular hemoglobin (MCH), packed cell volume (PCV), and mean corpuscular volume (MCV) remained unchanged. Immune and antioxidant parameters were also enhanced in fish fed the phytase-based feed, as evidenced by significant increases in alternative complement activity (ACH50), lysozyme activity, superoxide dismutase, and catalase, alongside reduced malondialdehyde concentrations (*P* < 0.05). Overall, these findings suggest that supplemental phosphorus (P) can be completely excluded from commercial tilapia feeds when the phytase enzyme (Ronozyme^®^HiPhos) is added at approximately 2000 FYT kg^− 1^. Phytase could also enhance the immune response and reduce the environmental impacts that would otherwise result from P release into the environment.

## Background

Fish meal (FM) has been traditionally used as the main protein source in the aquafeed industry, due to its excellent protein content, amino acid profiles, palatability, and digestibility. Despite the increasing demand for FM, the global production of FM has been gradually declining [1], presumably due to the decrease in the annual catch of forage fish, which are used for fishmeal and fish oil production [1]. This has created sharp competition for FM use by animal feed sectors, making FM the most expensive protein source in animal feed and aquafeed industries [2, 3]. On the other hand, the wide availability, accessibility, cost-effectiveness, and favorable nutritional profiles of plant protein sources make them an ideal candidate for FM replacement in aquafeed [2, 4]. However, these sources contain many antinutrients, including phytate (IP6), which is also known as phytic acid. IP6 is a heat-stable, phosphorylated form of myo-inositol, and is termed inositol polyphosphate. Phytate is an indigestible form of phosphorus (P) because up to 80% of P in plant-derived feeds is in phytate-bound form, rendering P unavailable to farmed aquatic animals [5]. Phytate chelates with P (and other positively charged ions, such as Ca, Fe, Mg, and Zn), thereby limiting their bioavailability [6]. It may also form insoluble complexes with cationic groups of proteins, amino acids, carbohydrates, and lipids in plant feedstuffs, making them less hydrolyzed by digestive enzymes [7–9]. For instance, Nile tilapia was reported to only digest 16% of the phytate-P intake [10]. Also, the phosphorus digestibility of a low-quality, plant-based diet fed to Nile tilapia was only 34.7% [11].

Phosphorus (P) is a vital element in many biological and metabolic processes in fish and other aquatic organisms. It is a fundamental component of bones, scales, cellular membranes, and nucleic acids, and is also engaged in cellular energy production [12, 13]. P is also required for the formation of organic phosphates, including DNA, phospholipids, coenzymes, and ATP. Thus, it is pivotal for the metabolism of proteins, lipids, and carbohydrates, thereby improving growth rates and nutrient utilization [14]. Additionally, P plays a key role in maintaining osmotic balance and supporting cell differentiation [15]. It further supports the fish’s immune system through enhancing phagocytic activity and the production of immunoglobulin antibodies [16].

Consequently, P is considered an essential component in fish and other aquatic animal feeds [12, 13]. P deficiency leads to reduced growth, poor feed digestibility and utilization, anorexia, lethargy, skeletal deformity, and immune retardation [12]. When Nile tilapia were fed a plant-based diet containing high phytate-P levels, they showed poor performance, feed efficiency, and nutrient digestibility [10, 11, 17]. Therefore, the demand for supplemental P in fish feeds has sharply increased, causing parallel increases in its prices [18]. Typically, inorganic P is included in the diets of herbivorous fish, such as tilapia and carps, mainly in the form of dicalcium phosphate (DCP) or monocalcium phosphate (MCP), which are produced from phosphate rock [17, 19]. However, phosphate rock is non-renewable and will be depleted if not wisely extracted and used [20]. In aquafeeds, phytase supplementation can be an ideal alternative for conserving phosphate rock.

Phytases are phosphatase enzymes that can hydrolyze the phytate molecule, making P available for absorption by fish, improving feed digestibility and absorption, and overall fish performance [21, 22]. Phytase can also release inositol (myo-inositol) through dephosphorylation of dietary phytate [22]. It takes part in many metabolic pathways in fish and other aquatic animals, including improving carbohydrate and lipid metabolism, modulating endocrine signaling within cells, stimulating the calcium release from the endoplasmic reticulum, and promoting immune responses [23, 24]. The efficiency of inositol release from phytate depends on the target species, the content of divalent cations, and the type, nature, and level of phytate [25]. On the other hand, inositol deficiency can cause hematological and pathological changes, including lethargy, reduced appetite, slow gastric emptying, poor growth, anemia, decreased intestinal immunity, fin erosion, poor lipid and carbohydrate metabolism, and lipid deposition in the liver [5, 26, 27]. Nonetheless, farmed aquatic animals are unable to secrete intestinal phytase. Consequently, a significant proportion of phytate-bound phosphorus is discharged into the water, exerting negative impacts on the aquatic environment [28]. As a result, supplemental phytase may become necessary for these farmed species, especially when they are fed plant-based feeds.

Nile tilapia (Family: Cichlidae) is now the second most globally cultured finfish species, after grass carp, yielding 5,300,000 mt in 2022 [1]. Tilapia farming is currently practiced in more than 120 countries, mainly in Asia, Africa, and Latin America [1]. Nile tilapia exhibit trophic plasticity in their feeding habits in the wild, feeding primarily on phytoplankton, periphyton, aquatic plants, bacterial films, and zooplankton, depending on fish size and developmental stage [2]. Under aquaculture conditions, commercial tilapia feeds are predominantly composed of plant-based ingredients that are high in phytate content, thereby increasing the demand for supplemental phosphorus.

The inclusion of phytases in tilapia feed formulations has thus emerged as a potential alternative because they can hydrolyze phytate and enhance the efficiency of plant-based feeds. Many studies showed that supplemental phytase in tilapia feeds, at ambient levels, improves fish performance and feed digestion and utilization [14, 28–31]. For example, phytase supplementation at 2000 FYT kg^− 1^ improved growth rates of Nile tilapia fed plant-based diets by 27.5% over the control diet [13]. It also enhanced feed efficiency, nutrient digestibility, and bone mineralization. Also, phytase inclusion in Nile tilapia feed at 1500 FTU kg^− 1^ enhanced the apparent digestibility of phosphorus by 289.83% (from 16.33% to 63.66%), calcium by 362.6% (from 9.33% to 44.26%), and other cationic minerals (Zn, Mg, Mn, Fe, and Cu) by 5.2–49.3%, hence optimizing mineral utilization [10]. More recently [17], demonstrated that supplementing Nile tilapia diets with 1000 FTU phytase kg^− 1^ improved P utilization and reduced the inclusion of exogenous P (dicalcium phosphate-DP) by up to 67% without compromising fish growth, feed utilization, and health status. These studies demonstrate that phytase can be used as an eco-friendly additive in Nile tilapia feeds, as it enhances the bioavailability of dietary phosphorus and other trace minerals [10, 13, 29], resulting in a notable reduction in P release into the aquatic environment.

Most of these investigations, however, were indoor trials conducted on early life stages, and for short durations, using lab-made test diets, although feed processing can significantly affect feed digestion, absorption, and bioavailability. These trials have also investigated the use of exogenous phytase in tilapia diets from performance perspectives. Nonetheless, limited information is available on the economic feasibility of phytase inclusion in tilapia feeds. As concluded by [17], future studies are needed on the effects of supplemental phytase and phosphorus on the fattening-finishing phases of tilapia production. Accordingly, this work evaluates the role of liquid phytase in growth rates, body composition, feed efficiency, digestive and liver function enzymes, and economic return in Nile tilapia fed commercial extruded diets under commercial farming conditions.

## Materials and methods

### Farming system and conditions

**Fig. 1:**
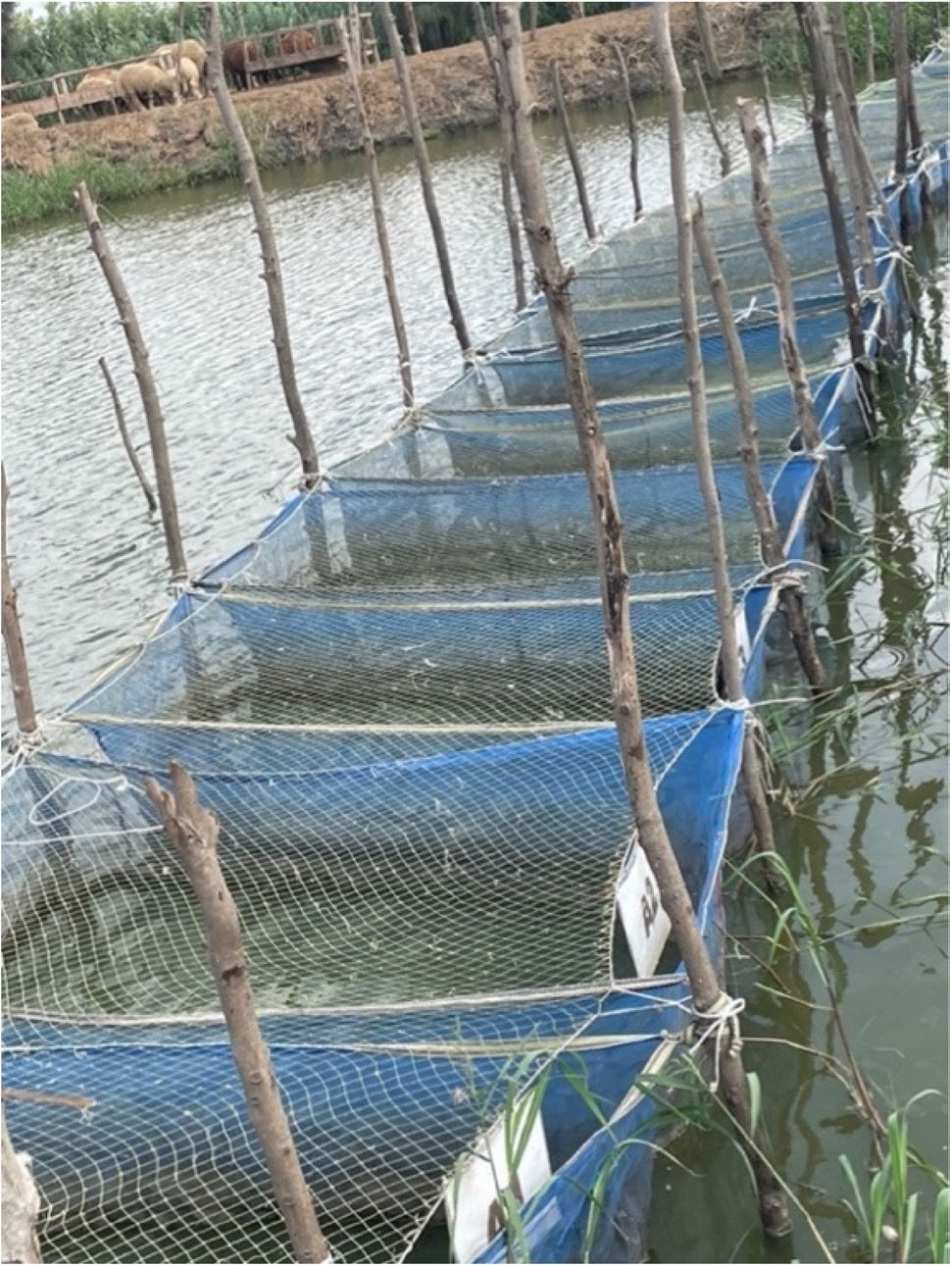
The hapas that were used for the trial

This research was executed in 3-m^3^ hapas (2 × 1 × 1.5 m, L × W × H), with a mesh size of 10 mm, and a water holding capacity of 2 m^3^ (Fig. 1). The hapas were installed in an earthen fishpond in Edku Province, Behaira Governorate, Egypt. Healthy all-male Nile tilapia (*Oreochromis niloticus*), with an average weight of 75.28 ± 0.66 g, sourced from the tilapia farm in which the feeding experiment was executed, were stocked in the hapas at a density of 40 fish per hapa. The formulated test diets were provided to each hapa at 3% of fish biomass per day, twice a day (08:00 and 15:00 h), for a 7-day acclimation phase. After acclimation, the fish in each hapa were netted, counted, and weighed, and the mean starting weight (g fish⁻¹) was recorded. Additional details on the culture system are available in a previously published study [32].

There was no water exchange throughout the trial; only seepage and evaporation losses were replaced. Additional paddle-wheel aeration was used regularly, especially during the night and early morning, to increase dissolved oxygen (DO) concentrations and reduce ammonia levels. The natural photoperiod cycle (about 12 h light: 12 h dark) was also adopted during the feeding trial. Water quality parameters underwent regular checks, measuring temperature, pH, dissolved oxygen (DO), total ammonia nitrogen (TAN) (NH_4_-N, mg L^-1^), unionized ammonia nitrogen (UAN) (NH_3_-N, mg L^-1^), nitrite (NO_2_), and nitrate (NO_3_). Surface water temperature and pH were tested twice daily (early morning and late afternoon) via portable pH and temperature testers (Hanna Instruments, Nusfalau, Romania). DO was also determined twice a day (early morning and late afternoon) with an EcoSense DO200A DO Probe (YSI Inc., Yellow Springs, OH, USA). TAN, NO_2_, and NO_3_ levels were quantified weekly (at mid-day) spectrophotometrically, using a YSI 9300 photometer (YSI Inc., Yellow Springs, OH, USA). Unionized ammonia–nitrogen (NH₃–N) concentrations were calculated from previously estimated TAN, water temperature, and pH values, following the method of [33]. The ranges and means (in parentheses) of tested parameters throughout the trial were as follows: temperature, 22–27 (24.45) ◦C; DO, 4.50–5.87 (4.54) mg L^-1^; NH_4_–N, 0.39–2.41 (0.79) mg L^-1^; NH₃–N, 0.01–0.13 (0.066) mg L^-1^; NO_2_, 0.021–0.044 (0.026) mg L^-1^; NO_3_, 0.11–0.27 (0.16) mg L^-1^; and pH, 7.61–8.45 (8.17). It is noteworthy that the total ammonia concentrations in the fishpond were not optimal for Nile tilapia. However, this species can tolerate relatively high levels of unionized ammonia (up to 7.4 mg L^-1^) [34]. Meanwhile, the unionized ammonia concentrations recorded in this study (0.01–0.13 mg L⁻¹) fall within the tolerance range of Nile tilapia and were unlikely to have imposed significant stress on the fish.

### Experimental feeds and feeding protocol

In a previous laboratory trial [35], we tested the effects of graded levels of liquid phytase (Ronozyme^®^HiPhos, 20,000 FYT g^− 1^) (dsm-firmenich, Wurmisweg 576, 4303 Kaiseraugst, Switzerland), produced from *Aspergillus oryzae* (DSM 33699), on Nile tilapia fed a commercial, phosphorus (P)-free diet. The best growth performance, feed efficiency, and immune response were achieved at 2000 FYT kg^− 1^. This phytase concentration (2000 FYT kg^− 1^) was used in the present field trial. Three isoproteinic (30% CP), isocalorific (17 MJ kg^− 1^) extruded (floating) tilapia diets (3 mm diameter) were formulated and manufactured by Makkah Aquafeed Company, Kafr el Sheikh, Egypt. The first diet, which is routinely used in commercial tilapia production in Egypt, contained 10 g monocalcium phosphate (MCP) kg^− 1^ and served as a reference diet. The second diet was enriched with 2000 FYT Ronozyme^®^HiPhos kg^− 1^, without MCP supplementation, while the third diet contained 50% of both MCP and phytase (5 g MCP kg^− 1^ + 1000 FYT kg^− 1^) (Table 1).

**Table 1:** Ingredients and proximate composition of the test diets (on an as-fed basis)

Ingredients (g kg^− 1^)	Test diets:		
	10 g MCP kg^− 1^ (control)	5 g MCP + 1000 FYT kg^− 1^	2000 FYT kg^− 1^
Fishmeal (local, 57% cp.)^1^	30.00	30.00	30.00
Poultry byproduct meal (local, 55% cp.)^2^	75.00	75.00	75.00
Soybean meal (46% cp.)^3^	425.00	425.00	425.00
Wheat bran^4^	287.00	287.00	287.00
Rice bran^5^	115.00	115.00	115.00
Yellow corn^6^	42.70	47.70	52.70
Soybean oil^7^	6.00	6.00	6.00
Monocalcium phosphate (MCP)^8^	10.00	5.00	0.00
Phytase (Ronozyme^®^HiPhos) (FYT kg^− 1^)^9^	0.00	1000	2000
Mineral premix^10^	1.00	1.00	1.00
Vitamin premix^10^	1.00	1.00	1.00
Vitamin C^11^	1.00	1.00	1.00
Additives^12^	6.30	6.30	6.30
Proximate analyses (%)			
Moisture	8.51	7.83	8.92
Crude protein	30.12	30.50	30.33
Crude lipid	5.46	5.62	5.57
Ash	7.80	8.34	7.84
Crude fiber	4.20	4.09	4.16
NFE^13^	43.91	43.62	43.22
Arginine	2.40	2.41	2.43
Histidine	0.84	0.84	0.85
Isoleucine	1.44	1.46	1.46
Leucine	2.46	2.45	2.48
Lysine	1.99	2.01	2.02
Methionine	0.73	0.74	0.76
Phenylalanine	1.54	1.54	1.56
Threonine	1.19	1.20	1.22
Tryptophan	0.48	0.50	0.47
Valine	1.61	1.60	1.62
Total phosphorus	1.12	1.01	0.89
Phytate-phosphorus^14^	0.50	0.50	0.51
Free phosphorus	0.62	0.50	0.38
Gross energy (MJ kg^− 1^)^15^	16.82	16.93	16.79

^1^Gulf Fish Processing Company, Kafr el Sheikh, Egypt

^2^Alwatania Poultry, 6th of October City, Giza, Egypt

^3^Imported, Hany Trade, Shebin El Kom, Menoufia, Egypt

^4^Egyptian Swiss Milling, Borg El Arab, Alexandria, Egypt

^5^Alalamia 2000 for Rice Mills. Kom Hamada, Behaira, Egypt

^6^Imported- Makka for Import & Export, 6th of October City, Giza, Egypt

^7^Alitalia Company, Industrial Zone, Metoubes, Kafr el Sheikh, Egypt

^8^Timab-Tunisie, Gabes, Tunisia

^9^dsm-firmenich, Wurmisweg 576, 4303 Kaiseraugst, Switzerland. The enzyme was top-sprayed onto the extruded feeds after drying

^10^ArabMix, Borg El Arab, Alexandria, Egypt. IU or mg kg^− 1^: Vitamin A; 6,000 000 IU, Vitamin D3; 400,000 IU, Vitamin E; 60,000, Vitamin K3; 4,000, Vitamin B_1_; 4,000, Vitamin B_2_; 4,000, Vitamin B_3_; 30,000, Vitamin B_5_; 10,000, Vitamin B_6_; 3,000, Vitamin B_12_; 20,, Biotin; 75, Folic Acid; 1,500, Iron; 20,000, Manganese; 7,000, Copper; 5,000, Zinc; 30,000, Selenium; 1,000, and Cobalt; 150

^11^Sodium Calcium Ascorbyl-Phosphate, Adisseo Nutrition Animal S.L.U., Huesca, Spain

^12^Include: Sodium bicarbonate, calcium carbonate, sodium chloride, L-methionine, L-lysine, antitoxin, and emulsifier

^13^Nitrogen-free extract (NFE) = 100 - (moisture + crude lipid + crude protein ‏+‏ ash)

^14^Calculated from the International Aquaculture Feed Formulation Database (IAFFD) using the data available in the Feed Ingredient Composition Database (FICD) (https://app.iaffd.com/ficd).

^15^Gross energy, calculated based on 39.5, 23.6, and 17.2 KJ g^− 1^ for lipids, protein, and carbohydrates, respectively [5]

The test diets were produced as follows. The diet mixtures were ground through a 0.4 mm grinder (Yemmak, Balikesir, Turkey) and extruded through a 3 mm-diameter die in a single-screw extruder (Wenger X-165, Sabetha, KS, USA) at a temperature of 100 °C and a pressure of 35 BAR for 1.5–2.0 min. Produced pellets were dried at 100–120 ◦C in a dryer (Wenger X-165, Sabetha, KS, USA). The liquid phytase was thoroughly mixed with soybean oil, injected into a drum coater (Yangzhou, China), and top-sprayed onto the pellets after drying. The pellets were subsequently cooled to ambient temperature and packed in 25-kg polyethylene bags.

The feed was provided to the hapas at 3% of fish biomass per day, apportioned into two separate feedings (08:00 and 15:00 h), for 80 days. When the water temperature decreased to 24 °C, the daily ration was lowered to 2.5%. Fish were weighed at 10-day intervals, and the food allowance was recalculated. At the end of the field experiment, fish from each hapa were collected, counted, and weighed in bulk, and mortality rates were recorded. Growth performance, feed efficiency, body composition, economic return, digestive and hepatic enzyme activities, and immune and antioxidant status were assessed as described below. 

### Calculation of fish performance

Growth performance was calculated as follows:

- The percent weight gain (PWG) = 100 [W_f_ (g) –W_i_ (g) / W_i_ (g)],
- Specific growth rate (SGR, % day^− 1^) = 100 (Ln W_f_ – Ln W_i_) / *t*), where: W_i_ and W_f_ are the initial and final weights (g), and *t* is the time of trial (days).
- Feed conversion ratio (FCR) = dry feed intake (g) / fish live weight gain (g),
- Protein efficiency ratio (PER) = weight gain (g) / protein intake (g), and.
- Protein productive value (PPV) = 100 [protein gain (g)/protein fed (g)].

### Economic analysis

The economic evaluation involved calculating the total fish harvest, value of fish sales, feed costs, crop revenue, gross profit, and the cost-effectiveness of phytase-based diets compared with the reference diet. The following metrics were computed: total feed consumed (kg m^− 3^), total feed cost ($US m^− 3^), and total fish sales revenue ($US m^− 3^), applying the following equations, as outlined by [32] and [36].

- Cost of feed consumed ($US m^-3^) = Total feed consumed (kg m^-3^) x feed cost kg^-1^.
- Fish harvest (kg m^-3^) = Total fish harvest per hapa/2.
- Value of fish yield ($US m^-3^) = Total harvest (kg m^-3^) x wholesale price ($US kg^-1^).
- Gross earnings ($US m^-3^) = Value of fish yield – cost of feed consumed.
- Economic return (%) = 100 (earnings from phytase-based diets – earnings from the reference diet)/ earnings from the reference diet.
- Economic conversion ratio (ECR) = Cost of feed consumed ($US kg^-1^) x FCR.

### Body composition and phosphorus analyses

Once the feeding trial was finished, four fish from each hapa were frozen at -20 °C for subsequent body analyses. Additionally, 5 fish were preserved by freezing at − 20 °C before the commencement of the trial for initial body content assessment. The proximate composition of both experimental diets and fish muscles was assessed following the standard procedures [37].

Flesh samples were taken from 4 fish per hapa, frozen at -20 °C, and used for tissue phosphorus (P) analysis. These 4 fish were cooked in boiling water for about 15 min and left to cool at room temperature. The bones were then removed, cleaned of any flesh remains, dried at 100 °C for 6 h, and kept in labeled vials for subsequent P analysis. P analysis was run using Inductively Coupled Plasma-Optical Emission Spectroscopy (ICP-OES), as highlighted by [32].

### Digestive enzymes analysis

Following the completion of the field feeding experiment, a sample of four fish was selected from each hapa and allocated for analysis of digestive enzymes. They were anesthetized with clove oil (0.5–1.0 ml per litre) [32], after which the gastrointestinal tract was excised and rinsed thoroughly with chilled deionized water. Intestinal digesta were collected and homogenized in a phosphate-buffered saline solution (pH 7.5) at 4 °C. Following spinning of the resulting mixture at 5000 g for 20 min at 4 °C, the supernatant was carefully separated and stored at the same temperature. Intestinal protease, lipase, and amylase activities were quantified as previously pointed out [38, 39]. The Bradford technique [40] was adopted to determine the total soluble protein in diluted tissue homogenates, utilizing bovine serum albumin as a standard. Nα Benzoyl DL arginine p-nitroanilide was used for assessing the activity of trypsin [41]. Lipase activity was determined according to the method of [42], employing b-naphthyl caprylate as a substrate. One lipase unit was equivalent to the production of 1 mg β-naphthol minute^− 1^. Amylase activity was quantified utilizing soluble starch as a substrate in a reaction with 3, 5-dinitrosalicylic acid [43]. A spectrophotometer (UV2802S, Unico, China) was used to monitor changes in absorbance to ensure overall enzymatic activity. The activities of these enzymes were presented as U per mg protein.

### Hepatic function enzyme analysis

For hepatic enzyme analysis, livers were removed, under aseptic conditions, from anesthetized 4 fish per hapa, and frozen at -20 °C. The activities of alanine aminotransferase (ALT), aspartate aminotransferase (AST), and lactate dehydrogenase (LDH) were measured as outlined by [44]. The activity of ALT was assessed by tracking the increase in absorbance associated with pyruvate formation or the decrease in NADH absorbance as it was oxidized during the reaction. The activity of AST was measured by monitoring the rise in absorbance associated with oxaloacetate formation or the decrease in NADH absorbance resulting from its consumption during the reaction. LDH activity was assessed by monitoring the reduction in absorbance associated with NADH consumption or the increase in absorbance linked to pyruvate production. The activity of hepatic enzymes was reported as U per liter.

### Hematological analyses

For hematological and physiological analyses, six fish were randomly collected from each hapa, and anesthetized with clove oil (0.5–1.0 ml L⁻¹) [32, 45]. Blood was drawn from the caudal vein of four fish into serum red-cap tubes, using sterile syringes, and spun at 6000 RPM for 10 min. The resulting supernatant was transferred to 2-ml sterile Eppendorf tubes and kept at − 80 °C for biochemical assays. Blood from the remaining two fish from each hapa was collected into EDTA tubes for complete blood profile analysis. Hematological parameters were analyzed, including hemoglobin (Hb), total red blood cells (RBCs), total leukocytes (WBCs), lymphocyte percentage, monocyte percentage, eosinophil percentage, basophil percentage, and packed cell volume (PCV%) via Hematology Analyzer BC-20s (Mindray, Hong Kong, China).

### Immune and antioxidant enzyme analyses

The target immune and antioxidant parameters, including phagocytic activity, ACH50, lysozyme, superoxide dismutase (SOD), glutathione peroxidase (GPx), malondialdehyde (MDA), and catalase activities, were determined as follows. For measuring phagocytic activity (PA), 200-µL of leukocyte suspension was incubated in tubes with Amber-Lite (AL) medium, to which 100 µL of formalin-killed *Staphylococcus aureus* preparation (ATCC 25923- The Naval Medical Research Unit No. 3, Cairo, Egypt) was added. The suspension was spun at 3000 × g for 5 min at 4 °C, the supernatant discarded, and the remaining pellets were smeared onto clean microscope glass slides, air-dried and stained with Wright–Giemsa stain (Sigma-Aldrich, St. Louis, MO, USA). Phagocytic cells were counted microscopically, and the PA (%) was calculated as follows: PA (%) = 100 × (number of phagocytic cells / total number of leukocytes examined) [46].

Lysozyme (LSZ) activity assay was carried out using a colorimetric commercial kit (EnzChek™ Lysozyme Assay Kit- Catalog Number: E22013) (Thermo Scientific Portable Analytical Instruments Inc., Tewksbury, MA 01876, USA), as stated by [45]. Alternative complement activity (ACH50) was determined following the procedure of Oriol [47]. SOD activity was measured spectrophotometrically using DetectX^®^ Superoxide Dismutase (SOD) Activity Kit- Catalog Number: EIASODC. The [48] method was applied to fluorometrically measure the level of malondialdehyde (MDA) as described previously [45]. Glutathione peroxidase (GPx) activity was determined with Glutathione Peroxidase (GSH-Px) Activity Kit (Catalog number: EEA010), following the manufacturer’s instructions. Catalase (CAT) activity was assessed by Catalase Colorimetric Activity Kit (Catalog Number: EIACATC). In this process, the catalase reacts with hydrogen peroxide (H_2_O_2_) to produce water and oxygen. The unreacted H_2_O_2_ reacts with the probe, and the resulting product can be quantitatively measured calorimetrically at OD 570 nm.

### Statistical data analysis

The data of this study were subjected to a one-way analysis of variance (ANOVA). Duncan’s multiple range test was applied to compare means when the F-values were significant. The obtained results are shown as means ± standard error (SE) of three replicates (*n* = 3), and the values are considered significantly different at *p* < 0.05.

## Results

### Growth performance and profitability

The results of this study indicated that no notable statistical divergence was found (*p* > 0.05) in growth and feed efficiency parameters of Nile tilapia fed the test diets, namely feed conversion ratio (FCR), protein efficiency ratio (PER), and productive protein value (PPV), with phytase supplementation (Table 2). On the contrary, supplemental phytase notably increased (*p* < 0.05) the economic return over the reference, MCP-supplemented diet. Economic analysis revealed that phytase-based diets at 2000 FYT kg^− 1^ (without MCP supplementation) or 1000 FYT kg^− 1^ (with 5 g MCP kg^− 1^), respectively, were 10.87% and 3.01% more cost-effective (*p* < 0.05) than the control, phytase-free diet (Table 2).

**Table 2:** Growth performance and economic analysis (mean ± SE; *n* = 3) of Nile tilapia fed the experimental diets

Parameter	Test diets:		
	10 g MCP kg^− 1^ (control)	5 g MCP + 1000 FYT kg^− 1^	2000 FYT kg^− 1^
Average initial weight (g fish^− 1^)	76.63 ± 1.50^a^	74.86 ± 2.19^a^	75.25 ± 1.68^a^
Average final weight (g fish^− 1^)	196.82 ± 3.80^a^	189.83 ± 3.07^a^	194.53 ± 7.03^a^
Weight gain (g fish^− 1^)	120.19 ± 2.36^a^	114.57 ± 4.13^a^	119.28 ± 8.71^a^
% weight gain	155.54 ± 1.12^a^	153.21 ± 9.14^a^	158.75 ± 15.11^a^
SGR (% day^− 1^)	1.17 ± 0.006^a^	1.16 ± 0.04^a^	1.18 ± 0.08^a^
FCR	1.33 ± 0.03^a^	1.38 ± 0.06^a^	1.36 ± 0.04^a^
PER	2.49 ± 0.09^a^	2.38 ± 0.11^a^	2.42 ± 0.06^a^
PPV (%)	51.24 ± 1.18^a^	51.47 ± 2.07^a^	55.36 ± 3.12^a^
Economic analysis			
Production (kg m^− 3^)	3.92 ± 0.08^a^	3.79 ± 0.06^a^	3.89 ± 0.14^a^
Feed cost ($US m^− 3^)	3.51 ± 0.13^a^	3.13 ± 0.07^b^	2.99 ± 0.16^b^
Fish sales revenue ($US m^− 3^)	7.83 ± 0.15^a^	7.58 ± 0.12^a^	7.78 ± 0.28^a^
Gross profit ($US m^− 3^)	4.32 ± 0.09^a^	4.45 ± 0.10^a^	4.79 ± 0.13^b^
Profitability (%) relative to the control diet		3.01 ± 2.37^a^	10.87 ± 2.89^b^
Economic conversion ratio (ECR)	4.67 ± 0.02 ^a^	4.26 ± 0.03^b^	4.11 ± 0.02^c^

*MCP* monocalcium phosphate, *SGR* specific growth rate (% day^− 1^), *FCR* feed conversion ratio, *PER* protein efficiency ratio, and *PPV* protein productive value

Numbers within the same row sharing the same superscript letter do not significantly differ at *p* < 0.05

### Body composition

Dietary phytase exerted no statistically significant effect on proximate flesh composition (*p* > 0.05), except for ash content, which increased progressively with increasing phytase levels (*p* < 0.05) (Table 3). Additionally, the phosphorus (P) levels in the flesh and bones of Nile tilapia fed the phytase-based diets were higher (*p* < 0.05) than those observed in fish fed the MCP-based diet. Bone P concentrations consistently exceeded those in the flesh across all phytase concentrations. Consequently, P retention in both flesh and bones was significantly greater (*p* < 0.05) in fish receiving 1000 and 2000 FYT kg⁻¹ phytase than in those fed the MCP-supplemented diet.

**Table 3:** The composition of flesh (mean ± SE; *n* = 3), based on a wet weight, of Nile tilapia fed the experimental diets

Parameter (%)	Initial	Test diets:		
		10 g MCP kg^− 1^ (control)	5 g MCP + 1000 FYT kg^− 1^	2000 FYT kg^− 1^
Moisture	76.19	76.06 ± 0.27^a^	74.91 ± 0.41^a^	75.34 ± 1.29^a^
Crude protein	18.17	19.61 ± 0.39^a^	20.24 ± 0.52^a^	21.03 ± 0.68^a^
Crude lipid	3.19	3.38 ± 0.11^a^	3.46 ± 0.31^a^	3.57 ± 0.29^a^
Ash	1.23	1.29 ± 0.09^a^	1.47 ± 0.03^b^	1.53 ± 0.08^b^
Flesh P	0.91	1.00 ± 0.08^a^	1.08 ± 0.05^ab^	1.15 ± 0.11^b^
Flesh P retention (%)^1^		9.89 ± 1.06^a^	18.68 ± 0.78^b^	26.37 ± 1.34^c^
Bone P	6.02	6.67 ± 0.11^a^	7.17 ± 0.15^b^	7.31 ± 0.08^b^
Bone P retention (%)^1^		10.79 ± 0.56^a^	19.10 ± 1.03^b^	21.42 ± 1.67^b^

*MCP* monocalcium phosphate, *P* phosphorus

^1^P retention = 100 (Final P – Initial P)/Initial P

Numbers within the same row sharing the same superscript letter do not significantly differ at *p* < 0.05

### Digestive and hepatic enzyme activity

Data on digestive enzyme activities (amylase, lipase, and protease) are presented in Table 4. The results showed that replacing MCP with phytase did not significantly affect these activities (*p* > 0.05). Likewise, neither MCP supplementation nor phytase inclusion had a significant effect on hepatic enzyme activities (ALT, AST, and LDH) (Table 4).

**Table 4:** Digestive enzyme and liver function enzyme activities (mean ± SE; *n* = 3) in Nile tilapia fed the experimental diets

Enzyme	Test diets:		
	10 g MCP kg^− 1^ (control)	5 g MCP + 1000 FYT kg^− 1^	2000 FYT kg^− 1^
Amylase (U mg^− 1^)	22.21 ± 0.97^a^	23.75 ± 1.68^a^	20.42 ± 0.69^a^
Lipase (U mg^− 1^)	48.22 ± 1.41^a^	48.74 ± 1.32^a^	46.83 ± 1.47^a^
Protease (U mg^− 1^)	66.18 ± 1.89^a^	62.79 ± 2.61^a^	65.08 ± 1.37^a^
LDH (U L^− 1^)	132.15 ± 1.46^a^	134.52 ± 1.06^a^	130.30 ± 1.51^a^
AST (U L^− 1^)	13.33 ± 0.86^a^	12.13 ± 0.59^a^	14.33 ± 0.33^a^
ALT (UL^− 1^)	12.67 ± 0.88^a^	10.98 ± 0.34^a^	13.17 ± 1.45^a^

*ALT* alanine aminotransferase, *AST* aspartate aminotransferase, and *LDH* lactate dehydrogenase

Numbers within the same row sharing the same superscript letter do not significantly differ at *p* < 0.05

### Hematological parameters

The current findings indicate that phytase inclusion exerted statistically significant effects (*p* < 0.05) on erythrocyte counts (RBCs), hemoglobin level (Hb), and leukocyte counts (WBCs) (Table 5). The RBC counts, WBC counts, and Hb level increased notably (*p* < 0.05) in fish fed on the phytase-based feeds, compared with those offered the P-enriched diet. However, no statistically significant divergence (*p* > 0.05) was detected between fish fed the diet containing 5 g MCP + 1000 FYT kg^-1^ and those fed 2000 FYT kg^-1^ without phosphorus supplementation. Other hematological parameters (MCH, PCV, and MCV) were not significantly affected by dietary treatments (*p* > 0.05).

**Table 5:** Blood parameters (mean ± SE; n = 3) in Nile tilapia fed the experimental diets

Blood parameter	Test diets:		
	10 g MCP kg^− 1^ (control)	5 g MCP + 1000 FYT kg^− 1^	2000 FYT kg^− 1^
RBCs (10^6^ cells mm^− 3^)	2.26 ± 0.05^a^	2.52 ± 0.02^ab^	2.58 ± 0.08^b^
WBCs (10^3^ cells mm^− 3^)	173.85 ± 2.59^a^	194.33 ± 2.14^b^	192.03 ± 2.39^b^
Hemoglobin (g dl^− 1^)	11.09 ± 0.36^a^	12.97 ± 0.22^b^	13.26 ± 0.16^b^
MCH (pg)	50.63 ± 0.57^a^	51.32 ± 0.85^a^	52.17 ± 1.74^a^
PCV (%)	33.20 ± 1.41^a^	35.06 ± 2.54^a^	33.93 ± 0.52^a^
MCV (fl.)	146.67 ± 3.23^a^	144.43 ± 1.72^a^	139.73 ± 1.57^a^

*MCP* monocalcium phosphate, *RBCs* red blood cells, *WBCs* white blood cells, *MCH* mean cell hemoglobin, *PCV* Packed cell volume, *MCV* mean cell volume

Numbers within the same row sharing the same superscript letter do not significantly differ at *p* < 0.05

### Immune response and antioxidant status

**Fig. 2:**
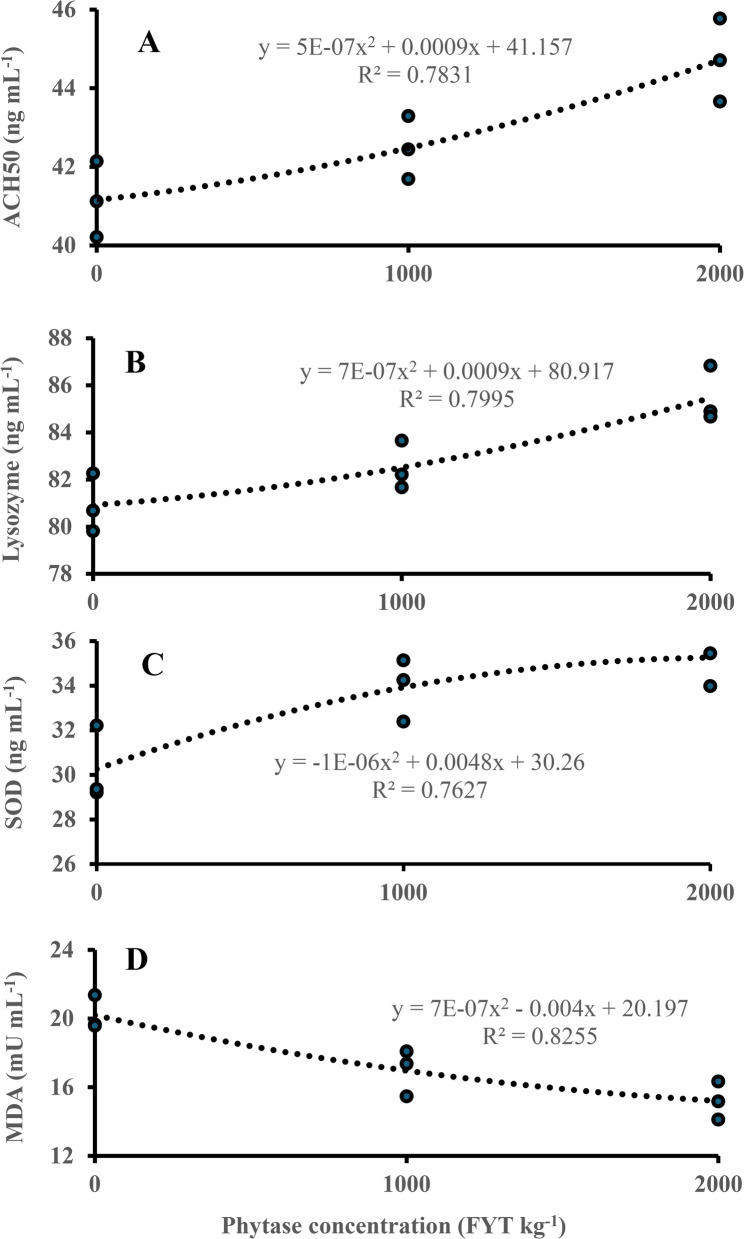
Effects of dietary phytase on **A**, ACH50 (alternative complement pathway); **B**, lysozyme; **C**, SOD (superoxide dismutase); and **D**, MDA (malondialdehyde) in Nile tilapia fed the test diets

Phytase enrichment significantly changed immunological and antioxidant parameters (*p* < 0.05), except for phagocytic activity (PA) and glutathione peroxidase (GPx), which were not significantly influenced (*p* > 0.05) (Table 6). Other parameters, including alternative complement pathway activity (ACH50), lysozyme activity, superoxide dismutase (SOD), and catalase, were notably higher (*p* < 0.05) in fish fed the phytase-based diets than in those offered the MCP-based diet (Fig. 2A-C). The all-phytase diet (without supplemental MCP) showed the highest immune and antioxidant values. An opposite trend was found in malondialdehyde (MDA) value, which was significantly reduced in fish fed the phytase diets (Fig. 2D).

**Table 6:** Immune response and antioxidant status (mean ± SE; *n* = 3) of Nile tilapia fed the experimental diets

Parameter	Test diets:		
	10 g MCP kg^− 1^ (control)	5 g MCP + 1000 FYT kg^− 1^	2000 FYT kg^− 1^
ACH50 (ng mL^-1^)	41.16 ± 0.72^b^	42.47 ± 0.46^b^	44.71 ± 0.61^a^
Phagocytic activity (µM mL^-1^)	1.21 ± 0.01^a^	1.28 ± 0.03^a^	1.33 ± 0.04^a^
Lysozyme (ng mL^-1^)	80.92 ± 0.56^b^	82.51 ± 0.59^ba^	85.49 ± 0.68^a^
Superoxide dismutase (mU mL^-1^)	30.26 ± 0.98^b^	33.93 ± 0.81^a^	35.26 ± 0.69^a^
Malondialdehyde (mU mL^-1^)	20.26 ± 0.65^b^	16.97 ± 0.78^ba^	15.20 ± 0.64^a^
Glutathione peroxidase (mU mL^-1^)	3.42 ± 0.09^a^	3.33 ± 0.13^a^	3.41 ± 0.12^a^
Catalase (mU mL^-1^)	507.40 ± 2.19^b^	506.23 ± 2.81^b^	524.23 ± 1.71^a^

*MCP* monocalcium phosphate, *ACH50* alternative complement pathway activity

Numbers within the same row sharing the same superscript letter do not significantly differ at *p* < 0.05

## Discussion

In Egypt, annual aquafeed production exceeds 1.4 million mt, of which approximately 90% consist of extruded feeds [49]. Over 95% of these feeds are derived from plant-based ingredients that are rich in phytate. Phytate strongly chelates phosphorus and other divalent minerals such as Ca, Fe, Mg and Zn, rendering them unavailable for the target fish [5, 6]. This interference with mineral bioavailability adversely affects growth performance, feed efficiency, skeletal mineralization, cellular metabolism, and energy transfer, as phosphorus is an essential structural component of bones, scales, nucleic acids, and phospholipids, and a key element of ATP‑mediated energy metabolism [12–15]. This implies that a large proportion of phytate-bound P is ultimately released into the environment. It has been reported that 60–85% of P inputs (mainly from the feed) in tilapia pond farms are discharged and accumulated in pond sediments [9, 50]. Prolonged and excessive release of inorganic P into the aquatic systems can also lead to significant adverse environmental impacts, such as water deoxygenation (hypoxia), algal blooming, and eutrophication, causing severe damage to the aquatic ecosystems [51]. Consequently, exogenous phosphorus (P) in the form of MCP and DCP is added to the feed formulation to compensate for the unavailable, phytate-bound P.

Dietary phytase supplementation has emerged as an effective strategy to alleviate these adverse effects by hydrolyzing dietary phytate, thereby increasing phosphorus bioavailability, and reducing phosphorus discharge into the environment while improving fish performance, nutrient digestibility, and overall fish health [10, 13, 17, 28, 31, 52]. In support, [53] found that phytase inclusion at 4000 FTU kg^-1^ significantly improved fish growth, feed digestibility, and P retention in cage-farmed Nile tilapia. At this phytase level, apparent P digestibility increased by 181% (from 29.2% to 82.1%) while body P increased by 109.8% (from 4.1% to 8.6%). P discharge into the water was also reduced by 76.5% (from 71% to 16.7%). In another study, P digestibility in Nile tilapia was increased by 172.7% when the diet was supplemented with 1000 FTU kg^-1^ phytase [54]. These findings clearly demonstrate that dietary phytase can significantly reduce P discharge through improving body P retention.

Due to their beneficial attributes, phytases have been widely used as a partial or total substitute for dietary P in tilapia feeds. Previous studies revealed that supplemental phytases, at varying levels, enhance growth, feed digestibility, and efficiency, and P bioavailability in Nile tilapia [31, 55–57]. For example, supplementing Nile tilapia diets with 1000–1500 FTU kg^-1^ reduced the need for exogenous P (DCP) by up to 67% [17], 75% [58], and 100% [59], without negatively affecting fish performance, feed utilization, and health status. Higher phytase levels (2000 FTU kg^-1^) were reported for optimum growth and feed efficiency of Nile tilapia fed P-free diets [35]. However, lower phytase level (660 FTU kg^-1^) was also found to support optimal growth, energy bioavailability, and carbohydrate metabolism in fish fed diets of plant origin [31]. These discrepancies in fish response to dietary phytase may stem from the differences in fish size, phytase source, form (liquid vs. granulated), dose, application method, diet composition, dietary P levels, and fish’s ability to consume plant feedstuffs [60–62].

In our field trial, exogenous P in commercial, plant-based Nile tilapia feed was completely replaced with 2000 FYT kg^-1^ phytase, without any adverse impacts. The growth performance, feed efficiency, and body constituents of fish receiving the all-phytase diet were comparable to those of fish fed the commercial, MCP-based diet. Moreover, fish fed a P-free diet, supplemented with 2000 FYT kg^-1^ phytase, exhibited increased ash and P contents in their flesh and bones, although these diets contained less available phosphorus than the P-rich diet. This may have stemmed from phytate hydrolysis by phytase, making P more readily available for absorption and retention [21, 22, 63]. Also, phytase‑mediated dephosphorylation of phytate releases myo‑inositol, a biologically active compound (phosphatidylinositol) with recognized roles in insulin signaling, carbohydrate and lipid metabolism, membrane phospholipid turnover, endocrine signaling, immune competence, and intracellular Ca²⁺ mobilization in fish [5, 13, 22, 26, 27]. On the other hand, inositol deficiency can lead to impaired growth, reduced appetite, fatty liver, anemia, and inhibited intestinal and immune functions [5, 26, 27]. Thus, the beneficial effects of phytase observed in our study are likely driven by a combination of improved mineral nutrition and inositol‑mediated metabolic regulation.

Unlike the above-mentioned studies, the current study was conducted as a field trial under commercial farming conditions, utilizing extruded commercial tilapia feeds. This experiment was also conducted on large-sized fish over a sufficient period to reach a final weight close to market size. This experimental design facilitated a more robust analysis and a comprehensive economic evaluation of the results. Thus, our study may likely be more reliable than these studies. It may also offer more practical relevance and reproducibility than previously mentioned studies.

Phytase supplementation can significantly affect the feed cost, profitability, and overall economic efficiency [59]. The cost-effectiveness of dietary phytase in aquafeeds is governed by the feed composition and quality, phytase source and cost, fish species, and feeding duration [31]. In the current trial, the economic analysis showed that phytase-based diets at 2000 FYT kg^-1^ (without P addition) or 1000 FYT kg^-1^ (with 5 g MCP kg^-1^), respectively, generated 10.87% and 3.01% more profit than the reference, phytase-free diet, although biological performance was similar among all diets. These economic benefits have been primarily attributed to reduced feed costs associated with phytase supplementation, along with improvements in animal performance. Similarly, [59] found that Nile tilapia fed 1500 FTU phytase kg^-1^, without DCP supplementation, attained significantly higher economic efficiency (109.7%) than those offered a DCP-based diet (82.07%), and a low-P diet (78.78%). However, the economic outcomes of that study may be of limited reliability, as it was conducted using fingerling fish, glass aquaria, and lab-made feeds. Likewise, the profit index of African catfish (*Clarias gariepinus*) fed phytase-based diets at 2000 FTU kg^-1^ was 10.5% more profitable than those offered a phytase-free diet [64]. Additionally, in rainbow trout (*Oncorhynchus mykiss*) fed graded levels of phytase (0–3000 OUT kg⁻¹), optimal economic efficiency was achieved at 750 OUT kg⁻¹, with a 46.9% increase in profitability compared with the control diet [9]. On the other hand, [65] demonstrated that in Nile tilapia receiving graded levels of phytases of fungal and/or bacterial origin, the phytase level that produced the poorest performance (500 and 1000 FTU kg^-1^) had the highest economic return. These authors concluded that the enhancement in growth performance may not be an accurate measure of economic performance. Collectively, these studies demonstrate that the cost-effectiveness of dietary phytase must be evaluated with careful consideration of feed composition and quality, phytase source and cost, fish species and size, feeding regimes, and farming systems.

In our study, the activity of digestive enzymes in Nile tilapia fed the phytase-based diets was similar to that of those fed the reference, DCP-rich diet. This may be attributed to phytate hydrolysis and the release of myo-inositol, which improves digestive capability, as demonstrated in obscure puffer (*Takifugu obscurus*) [66], juvenile Jian carp (*Cyprinus carpio*) [65], and Nile tilapia [67]. Phytase can also decompose phytin-protein complexes and neutralize the negative impacts of phytate on dietary protein in animal feed, thereby enhancing protein and amino acids digestion and utilization [68]. Moreover, phytase can improve gut health by increasing the intestinal villus length, width, and interspace, and goblet cell number, leading to significant enhancement of absorptive capacity in the intestinal villi and elevation in digestive enzyme activities in fish intestines [59, 65, 66, 69].

Phytase supplementation in the present study also significantly enhanced innate immune responses and antioxidant defenses, as evidenced by elevated lysozyme activity, phagocytosis, alternative complement activity (ACH50), and antioxidant enzymes (SOD and catalase), alongside reduced hepatic enzyme activities (AST, ALT, LDH). Phytase-based diets also increased RBCs, WBC counts, and blood hemoglobin levels. These immunomodulatory and antioxidant effects may be partly due to increased bioavailability of phosphorus and trace minerals, which are known to support phagocytic activity, antibody production, and redox balance [16]. Also, dietary phytases can enhance hematopoiesis, the biological process responsible for blood cell production [70]. Thus, these findings indicate that dietary phytase improved liver integrity, reduced oxidative stress, and enhanced cellular stability [71, 72]. Our results are consistent with previous findings in Nile tilapia [56, 59, 72–74], African catfish [14, 75], grass carp (*Ctenopharyngodon idellus*) [76], gibel carp (*Carassius auratus gibelio*) [69, 76], crayfish (*Procambarus clarkii*) [77], yellow catfish (*Pelteobagrus fulvidraco*) [78], and European seabass [36]. Overall, these outcomes demonstrate that supplemental phytase does not compromise fish health, even at low dietary P levels.

## Conclusion

The present findings confirm that dietary phytase acts not only as a phosphorus‑releasing enzyme but also as a nutritional modulator of mineral utilization, metabolic regulation, immune competence, antioxidant status, and economic profitability in Nile tilapia.

Supplementing the feed with 2000 FYT of phytase (Ronozyme^®^HiPhos) kg^− 1^ can totally replace exogenous inorganic phosphorus without compromising fish performance, physiological functions, and health status, while increasing economic return. Also, phytase markedly reduces phosphorus discharge into aquatic environments, thereby mitigating its adverse impacts on aquatic ecosystems.

## Data Availability

Data from the study are available from the corresponding authors upon reasonable request.

## Abbreviations

ACH50: Alternative complement activity
ALT: Alanine aminotransferase
AST: Aspartate aminotransferase
DO: Dissolved oxygen
ECR: Economic conversion ratio
EDTA: Ethylenediaminetetraacetic acid
FCR: Feed conversion ratio
GPx: Glutathione peroxidase
Hb: Hemoglobin
MCH: Mean cell hemoglobin
MCP: Monocalcium phosphate
MCV: Mean cell volume
MDA: Malondialdehyde
NADH: Nicotinamide Adenine Dinucleotide, reduced form
PA: Phagocytic activity
PCV: Packed cell volume
PER: Protein efficiency ratio
PPV: Protein productive value
RBCs: Red blood cells
ROI: Return on investment
SGR: Specific growth rate
SOD: Superoxide dismutase
WBCs: White blood cells
